# On the Role of Glutamate in Presynaptic Development: Possible Contributions of Presynaptic NMDA Receptors

**DOI:** 10.3390/biom5043448

**Published:** 2015-12-14

**Authors:** Karlie N. Fedder, Shasta L. Sabo

**Affiliations:** Departments of Pharmacology and Neuroscience, Case Western Reserve University School of Medicine, Cleveland, OH 44106, USA; E-Mail: knf13@case.edu

**Keywords:** glutamate, NMDA, presynaptic, synaptogenesis, synapse formation

## Abstract

Proper formation and maturation of synapses during development is a crucial step in building the functional neural circuits that underlie perception and behavior. It is well established that experience modifies circuit development. Therefore, understanding how synapse formation is controlled by synaptic activity is a key question in neuroscience. In this review, we focus on the regulation of excitatory presynaptic terminal development by glutamate, the predominant excitatory neurotransmitter in the brain. We discuss the evidence that NMDA receptor activation mediates these effects of glutamate and present the hypothesis that local activation of presynaptic NMDA receptors (preNMDARs) contributes to glutamate-dependent control of presynaptic development. Abnormal glutamate signaling and aberrant synapse development are both thought to contribute to the pathogenesis of a variety of neurodevelopmental disorders, including autism spectrum disorders, intellectual disability, epilepsy, anxiety, depression, and schizophrenia. Therefore, understanding how glutamate signaling and synapse development are linked is important for understanding the etiology of these diseases.

## 1. Introduction

The molecular and cellular mechanisms by which genes and the environment interact to control circuit formation and refinement remain incompletely understood. While it is well established that sensory and neuronal activities induce structural remodeling of postsynaptic spines, axonal boutons also undergo activity-dependent growth and remodeling. Less is known about this process, but recent studies have provided important new insight. Therefore, this review will focus on activity-dependent control of presynaptic terminal development.

**Figure 1:**
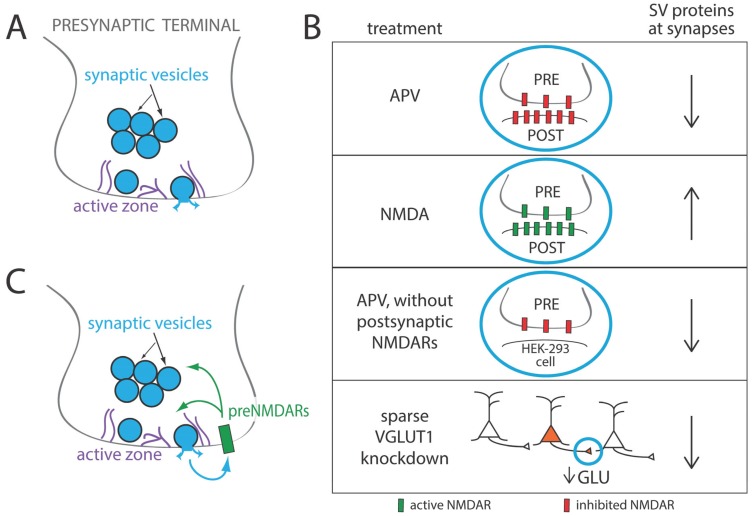
(**A**) Presynaptic terminals (or boutons) are specialized structures that form within axons at sites of synaptic contact with postsynaptic partners. Presynaptic terminals contain clusters of small synaptic vesicles that are loaded with small molecule neurotransmitters, such as glutamate and GABA. When an action potential invades the presynaptic terminal, synaptic vesicles fuse with a region of the plasma membrane known as the active zone, and a neurotransmitter is released into the synaptic cleft where it can bind to its receptors. Synaptic transmission occurs upon binding of receptors within the postsynaptic membrane; however, presynaptic neurotransmitter receptors also exist. The probability that a vesicle fuses in response to an action potential is known as the probability of release, and the postsynaptic response induced by fusion of a single vesicle is referred to as a miniature excitatory postsynaptic current, or mEPSC; (**B**) Presynaptic development is regulated by NMDA receptor activation, even when presynaptic terminals are formed with postsynaptic partners that lack NMDA receptors. Treatment of cortical neurons with APV, an NMDA receptor antagonist, reduced accumulation of synaptic vesicle and active zone proteins at developing synapses, while treatment with the NMDA receptor agonist NMDA did the opposite [14]. When cortical neurons formed presynaptic terminals with non-neuronal HEK-293 cells that express neuroligin but not NMDARs, treatment with APV reduced synaptic vesicle protein content at these terminals. When VGLUT1 was knocked down in individual cortical neurons in culture to reduce synaptic glutamate release, presynaptic terminals formed by those neurons had reduced synaptic vesicle protein content. This indicates that presynaptic development is regulated by glutamate release in a cell-autonomous manner [15]; (**C**) Based on these observations, we hypothesize that presynaptic NMDA receptors may facilitate presynaptic development.

Pioneering work from a number of laboratories has begun to identify the cellular events in presynaptic terminal formation. In general, presynaptic terminal formation is induced by axo-dendritic contacts [1,2,3,4,5,6]. Upon stabilization of an axo-dendritic contact, synaptic vesicle (SV) and active zone (AZ) proteins are accumulated at the site of contact [1,2,3,4,5,6]. Recruitment of presynaptic proteins and structures begins rapidly, with the first components arriving on a time-scale of minutes [7,8]. In addition, specialized presynaptic structures, such as the AZ and a cluster of SVs are established at the site of contact (Figure 1A). Formation of the AZ is thought to be initiated by the fusion of AZ protein transport vesicles with the axonal surface [9]. SVs form within the nascent terminal or are acquired from preassembled clusters of SVs that are mobile within axons [7,10,11]. As a bouton continues to develop, the number of SVs within the terminal increases and the AZ expands, requiring continual recruitment of SV and AZ proteins [12,13].

Recently, we found that NMDA receptors (NMDARs) bi-directionally regulate the accumulation of SV and AZ proteins at nascent excitatory presynaptic terminals [14]. We also observed this NMDAR-dependent regulation at presynaptic terminals that did not have NMDAR-expressing postsynaptic partners (Figure 1B), and at terminals of individual neurons with impaired vesicular glutamate release in an otherwise active network (Figure 1B and [14,15]). These observations led us to hypothesize that presynaptic terminal development is facilitated by the activation of NMDARs in a cell-autonomous manner, without the need for retrograde signaling from postsynaptic partners (Figure 1C). It is not yet clear where the relevant NMDARs are localized, but one intriguing possibility is that glutamate regulates presynaptic development through local activation of presynaptic NMDARs (preNMDARs). Below, we will summarize our current understanding of preNMDARs and discuss their potential role in presynaptic development.

## 2. General Properties of NMDA Receptors

NMDARs are ionotropic glutamate receptors. Activation of NMDARs, and in particular calcium influx through their channels, plays critical roles excitatory synaptic transmission and synaptic plasticity. NMDARs are tetramers containing two GluN1 subunits co-assembled with GluN2 and/or GluN3 subunits. There are four GluN2 subunits (A through D) and two GluN3 subunits (A and B). While GluN1 is required for assembly of functional receptors, GluN2 subunits are necessary for glutamate-dependent activation: glutamate binds in the pocket created by the GluN2 amino terminal extracellular domain and the extracellular loop. Receptors that contain GluN2A and/or GluN2B subunits are typically blocked by magnesium unless they are activated in conjunction with depolarization, while incorporation of GluN2C/D or GluN3A/B subunits confers a low sensitivity to blockade by magnesium [16,17].

GluN2B (also known as GRIN2B or NR2B) is essential for proper neural development. During development, GluN2B is widely expressed and the predominant GluN2 subunit in the cortex and hippocampus. GluN2B homozygous knockout mice die around birth, and genetically swapping GluN2B with GluN2A results in poor survival, reduced NMDAR current amplitude, abnormal homeostatic plasticity, and abnormal social behavior [18]. At around 2–3 weeks postnatal, NMDARs in the cortex and hippocampus undergo a developmental switch in their subunit composition: GluN2B levels plateau, while GluN2A levels dramatically increase, particularly within postsynaptic densities [19]. In the adult, GluN2B is expressed specifically in the forebrain, where it is found in most cortical neurons, while GluN2A is expressed throughout the central nervous system (CNS) [20].

GluN2C/D and GluN3A/B subunit expression is also temporally and spatially regulated. In the rodent cerebral cortex, GluN2D is expressed during postnatal development, but only at modest levels when compared to the brainstem [21]. GluN2C expression is first detected during the first postnatal week, and its expression is high in the adult cerebellum [19]. GluN3A is expressed at high levels throughout the forebrain, midbrain, and cerebellum during postnatal development but is down-regulated in adults, while GluN3B expression is highest in adults [17].

Activation of NMDARs requires binding of a co-agonist, either glycine or D-serine, to the GluN1 subunit [22,23]. There is abundant evidence that supports a role for endogenous glycine and D-serine in regulating NMDAR function *in vivo* [22,23]. Recent evidence suggests that whether particular NMDARs utilize glycine or D-serine may be dependent on the type of synapse examined [24] or the localization of the receptors [25]. Both D-serine and glycine are already found in the CNS at birth and remain present throughout postnatal development [24,26,27,28,29]; therefore, either or both could be important for NMDAR activation at developing synapses.

## 3. Regulation of Presynaptic Development by Glutamate and Synaptic Activity

The role of glutamate and glutamate receptors in presynaptic development is both complex and controversial. Although presynaptic terminals can form in the absence of synaptic transmission [30,31], it is clear that activity shapes synapse development. Activity-dependent changes in synapse density and morphology and the mechanisms of those changes appear to vary with: (i) developmental age; (ii) duration of treatment; (iii) how activity is blocked (e.g., blocking action potentials *vs.* all synaptic activity); (iv) whether activity is modified globally or locally; and possibly (v) neuron type [14,32,33,34,35,36,37,38,39].

In developing neurons, the reported effects of synaptic activity on morphological development of presynaptic terminals have been variable. In one study, when synaptic glutamate release was severely impaired due to knockout of VGLUT1, presynaptic terminals contained fewer SVs and levels of SV proteins were reduced [40]. On the other hand, synaptic silencing increased AZ length [31]. Mice which lack transmitter release due to the genetic knockout of Munc18-1, a protein that is essential for synaptic vesicle exocytosis, have decreased synapse density, a smaller percentage of synapses with docked SVs, and a reduced number of SVs per synapse [41]. Blocking action potential-driven activity with TTX increased the size of the readily releasable pool of SVs in young (8–9 DIV) neurons [32]. It is worth noting that in each of these studies, observed changes could have been a result of overall changes in network excitability.

In general, in more mature neurons (at least 14 DIV for cultured neurons or postnatal day 14 *in vivo*), the chronic blockade of action potential (AP) driven synaptic activity throughout the network results in an increase in probability of release and mEPSC frequency without a change in synapse density [33,35,36,42,43,44,45,46]. In contrast, decreasing AP generation in individual neurons decreases mESPC frequency and the size and density of presynaptic terminals formed with the silenced neuron [47], though it is not known how silencing affects the presynaptic terminals of the silenced neuron.

Recently, we showed that knockdown of VGLUT1 in individual cortical neurons results in cell-autonomous reductions in the synaptic expression of both SV and AZ proteins [15]. In this case, it is unlikely that the observed effects were due to changes in network activity since VGLUT1 was knocked down in less than 1% of neurons. In addition, it is unlikely that these effects were due to altered action potential generation in either the knockdown neuron or its postsynaptic partner since (i) loss of VGLUT1 selectively affects glutamate release from the presynaptic terminals of the knockdown neuron and (ii) the postsynaptic neurons still receive normal input from the vast majority of their presynaptic partners. This observation points to a specific role of glutamate signaling during development of presynaptic terminals. These data are also consistent with the hypothesis that presynaptic glutamate receptors are involved in this regulation.

Interestingly, activity-dependent changes in synapse development and plasticity can be driven by spontaneous synaptic drive, independent of action potentials [48,49,50,51,52,53]. For example, control of synapse development by neuroligin and LRRTM2 depends on neuronal activity [54,55], and spontaneous activity is sufficient [38]. In developing neurons, blockade of spiking leads to either a reduction or no change in synapse density, possibly dependent on the duration of treatment [14,32,33], while blockade of both spontaneous and evoked transmitter release in young neurons appears to consistently cause a decrease in synapse density [41,56]. Interestingly, spontaneous and evoked glutamate release can activate distinct populations of NMDA receptors, even at the same synapses [57]. They can also drive different downstream signaling pathways that lead to unique effects on animal behavior [37,38,48,58,59].

## 4. Regulation of Synapse Formation by NMDA Receptors

Most studies on the effects of NMDARs on synapse formation and plasticity have been performed on relatively mature neurons, after the peak of synaptogenesis. In general, studies of mature synapses have shown that NMDAR blockade tends to decrease spine density or shift spine morphology to more filopodial-like structures [39,43,60,61,62,63,64,65,66,67], but this may not always correlate with a decrease in presynaptic terminal density [39,64]. Importantly, several reports suggest that NMDAR activity is important for presynaptic development and maturation [14,39,63,67,68,69]. At retino-collicular synapses, NMDAR blockade from birth increases synapse density in 6–10 day old animals but not in 14 day olds, while NMDA decreases synapse density at P14 but not P8 [61]. In young, cultured cortical neurons, 24–48 h exposure to NMDAR antagonists results in a reduction in the amount of SV and AZ proteins at individual synapses, while activation of NMDARs does the opposite [14]. In this case, no changes in synapse density are observed.

Differences in NMDAR-dependent regulation of presynaptic terminal development might arise from developmental changes in synaptogenic mechanisms. For example, immature synapses are highly sensitive to actin disruption, while mature synapses are resistant to actin-depolymerizing drugs [70]. Similarly, disruption of adhesive properties of cadherins leads to instability of immature, but not mature, synapses [71,72]. Finally, immature presynaptic terminal activity and synapse elimination are sensitive to protein synthesis inhibitors, while mature presynaptic terminals are fairly resistant to protein synthesis inhibitors [72].

NMDARs could signal cell-autonomously to alter presynaptic terminal development. Alternatively, activation of NMDARs on postsynaptic neurons could induce the release of a retrograde signal that in turn acts on the presynaptic partner to regulate its development. With regard to cell-autonomous effects of NMDARs, one report showed knockdown of GluN1 decreased mEPSC frequency in the hippocampus, and this was not dependent on NMDAR activity [60]. On the other hand, another study showed that Cre-mediated elimination of GluN1 in the cortex did not alter mEPSC frequency within GluN1-lacking neurons [39]. Both studies were performed on neurons >14 DIV, and neither study examined the effects of cell-autonomous removal of NMDARs on presynaptic development in GluN1-deficient neurons.

## 5. Presynaptic NMDA Receptors

The role of presynaptic ionotropic glutamate receptors at glutamatergic synapses has only recently been appreciated. Presynaptic NMDA receptors were first identified in cortical presynaptic terminals by electron microscopy by Aoki and colleagues in 1994 [73], while the first physiological evidence for preNMDARs in the cerebral cortex appeared in 1996 [74].

Although many studies report only postsynaptic NMDARs, both immunoperoxidase and immunogold electron microscopy studies have demonstrated NMDAR subunit immunoreactivity in axons and presynaptic boutons at a variety of synapse and neuron types. Several studies have used immuno-electron microscopy to demonstrate that NMDARs can be observed in presynaptic terminals in the cortex [73,75,76,77,78] and hippocampus [78,79,80,81], although there is some inconsistency in the details of (i) whether they are present at excitatory synapses; (ii) where they are localized within terminals; and (iii) how abundant this labeling is [75,76,77,78,79,80,81,82].

Additional evidence supports the existence of preNMDARs. Using ultrasynaptic fractionation, we showed that presynaptic NMDARs that contain GluN1 and GluN2B are present within the AZ membrane [83], where SV fusion and subsequent glutamate release occurs. At the light microscopic level, NMDARs have been examined in axonal growth cones of young hippocampal neurons, with varying results [84,85,86,87]. Using fluorescence imaging, we recently demonstrated that GluN1 is clustered at many (but not all) presynaptic terminals of cultured cortical neurons [83]. GluN1 puncta occur at a lower density in axons than dendrites [83] and are smaller and dimmer than dendritic GluN1 puncta in the same neuron. Presynaptic clustering of GluN1 occurs early in synapse development [83]. The early appearance of GluN1 at presynaptic terminals is consistent with a role for presynaptic NMDARs in synapse development.

In developing neurons, NMDARs located within presynaptic neurons are responsible for acute regulation of presynaptic plasticity. NMDARs in presynaptic neurons are essential for LTP at cortico-striatal synapses [88]. Within the cortex, activation of NMDARs in presynaptic neurons can facilitate spontaneous and evoked neurotransmitter release and is required for induction of timing-dependent LTD and pattern-dependent LTD [74,76,89,90,91,92,93,94]. The responsible receptors appear to contain GluN2B and GluN3A [21], but their subcellular localization was unclear until recently.

Recent evidence suggests that, at least at some synapses, preNMDARs are indeed responsible for presynaptic plasticity. An elegant study showed that axonal, but not dendritic, NMDARs are necessary for the induction of timing-dependent LTD in cortical layer 4-layer 2/3 synapses [93,94]. These effects of presynaptic NMDARs are observed at layer 4-layer 2/3 synapses but not at layer 2/3-layer 2/3 synapses formed with the same postsynaptic neuron [94]. In the cerebral cortex [91] and hippocampus [79], calcium transients can be imaged in presynaptic boutons upon focal uncaging of NMDA or glutamate. In addition, activation of NMDARs in a given neuron selectively regulates glutamate release at a subpopulation of synapses formed by that neuron [91]. This observation argues that the relevant NMDARs are activated locally, at or near presynaptic terminals.

Interestingly, preNMDARs appear to be restricted to a subset of synapses formed by the same neurons. In the cerebral cortex, layer five pyramidal neurons form presynaptic terminals with a range of postsynaptic partners, including other cortical pyramidal neurons, somatostatin-positive Martinotti cells (a type of GABAergic interneuron), and parvalbumin-positive basket cells (another type of GABAergic interneuron). Buchanan and colleagues showed that synapses formed between two pyramidal neurons and those formed between pyramidal and Martinotti neurons posses functional presynaptic NMDARs [91]. However, functional preNMDARs were only found at a subset of synapses formed between pyramidal neurons and parvalbumin-positive GABAergic neurons. Those synapses that appeared to have preNMDARs were formed with a distinct subtype of parvalbumin-positive cells that were defined by a unique axonal morphology [91].

**Figure 2:**
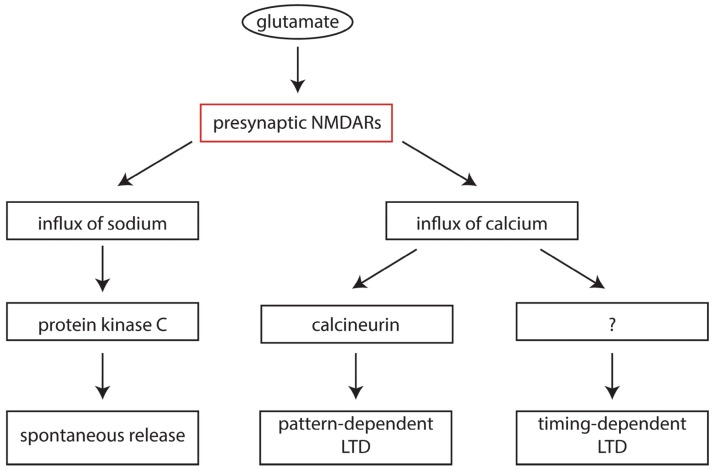
Signaling mechanisms downstream of presynaptic NMDA receptor activation. Presynaptic NMDARs control spontaneous glutamate release, timing-dependent LTD and pattern-dependent LTD through three distinct signaling pathways. Facilitation of spontaneous release does not depend on calcium influx. Timing-dependent LTD appears to require coincident activation of presynaptic CB1 receptors and glutamate release from astrocytes.

The down-stream mechanisms by which preNMDARs regulate presynaptic plasticity are not yet clear. PreNMDARs can pass calcium and some of their roles may depend on this calcium influx [74,79,91,95,96,97,98]; however, this is not always the case (Figure 2). Surprisingly, preNMDAR-dependent facilitation of spontaneous neurotransmitters does not require calcium influx from extracellular space or the release of calcium from intracellular stores [99]. It does depend on extracellular sodium, probably to depolarize the presynaptic terminal, but other roles for sodium influx are possible [99]. In addition, preNMDAR-dependent regulation of spontaneous glutamate release depends on protein kinase C (PKC), but the relevant phosphorylated target(s) of PKC have not yet been identified [99].

Presynaptic regulation of LTD may utilize distinct down-stream mechanisms (Figure 2). Timing-dependent LTD appears to depend on postsynaptic metabotropic glutamate receptors, coincident activation of presynaptic CB1 receptors and preNMDARs, and astrocyte release of glutamate [89,100,101,102]. In contrast, pattern-dependent LTD does not require metabotropic glutamate receptors, CB1 receptors, glia, postsynaptic calcium, or G-protein signaling [103]. However, pattern-dependent LTD does depend on the activity of presynaptic calcineurin [103].

Since NMDARs require a co-agonist for activation, it would be interesting to know whether preNMDARs are co-activated by glycine, D-serine, or both. GluN2B-containing NMDARs have a higher affinity for glycine than GluN2A-containing receptors, while GluN2B-containing NMDARs have a lower affinity for D-serine than NMDARs with GluN2A [22]. This suggests that glycine may be the preferred co-agonist of preNMDARs; however, either or both co-agonists could contribute to preNMDAR activation.

## 6. Presynaptic NMDA Receptors in Development

The role of axonal NMDARs appears to be developmentally regulated. NMDARs are observed in axons and axonal growth cones of hippocampal neurons during the first postnatal week, but expression in distal axons and growth cones is dramatically decreased later in postnatal development [83,84,85,86,87]. Similarly, ultrastructral evidence in the visual cortex indicates that the fraction of presynaptic terminals with identified NMDARs decreases significantly after the critical period for receptive field plasticity [76]. In addition, there is no apparent effect of preNMDARs on neurotransmitter release after postnatal day 23 [76], and the presence of NMDARs in biochemically isolated presynaptic membranes is reduced in mature animals when compared to young animals [83].

Since the period of highest NMDAR expression coincides with the period of intense synapse formation and an elevated probability of release has been proposed to enhance synapse formation, it has been hypothesized that activation of axonal NMDARs might facilitate synapse formation [104]. However, this hypothesis has not yet been directly tested. Formation of an individual synapse occurs over a time course of hours, and initial synaptogenesis occurs over a period lasting for several weeks in rodents, but the effects of chronic activation or inactivation of preNMDARs have not yet been studied. This is largely due to the difficulty of differentiating presynaptic and postsynaptic effects and because there is no method for selectively inhibiting, activating, or removing preNMDARs without also affecting postsynaptic NMDARs.

If preNMDARs were involved in synaptogenesis, activation of preNMDARs would provide a feedback mechanism that could ensure that functional presynaptic terminals are preferentially made and maintained, even in the absence of postsynaptic responses. PreNMDAR-dependent regulation of presynaptic bouton development could be especially important during circuit and sensory map development since many synapses in the early postnatal cortex are postsynaptically silent [66,105,106]. In contrast, preNMDARs are thought to be active [104,107], possibly due to depolarization of the presynaptic terminal.

Recent studies have demonstrated that preNMDARs are regulated by sensory input in both the somatosensory [108] and visual [109] cortex, providing an intriguing possible link between experience and synapse and circuit development. The converse may also be true: control of presynaptic development by glutamate and NMDARs could ultimately play a role in establishing receptive field properties. For example, coincident with the period of intense synapse formation and high expression of preNMDARs in the visual cortex [76], several important aspects of visual cortical receptive fields are established, including retinotopy, ocular dominance, and orientation selectivity. Glutamatergic signaling via NMDARs contributes to the establishment of these receptive field properties [110,111,112].

## 7. Relevance to Neurodevelopmental Disease

Impaired synapse formation and elimination is thought to contribute to pathogenesis of numerous neurodevelopmental and psychiatric disorders, including autism spectrum disorders (ASD), intellectual disability (ID), epilepsy, anxiety, depression, and schizophrenia [113]. For example, ASD is typically diagnosed before age three, during a period of intense synapse formation [114,115]. Analysis of human genetics, postmortem brain histopathology, and mouse models have all pointed to a role for synapse formation in ASD pathogenesis [114,116,117,118,119,120,121,122,123]. In addition, early life emotional experience both correlates with altered synapse density [124,125] and predicts anxiety and depression later in life [126,127,128]. Therefore, the likelihood of developing anxiety disorders has been proposed to be established during development of neural circuits [129].

Abnormal synaptic glutamate signaling has also been proposed to contribute to ASD, ID, epilepsy, schizophrenia, and depression. Mutations in NMDAR subunits have been linked to pathogenesis of several neurodevelopmental diseases—including ASD, ID, schizophrenia, and epilepsy [116,117,130,131,132,133,134]. Furthermore, changes in glutamate signaling and abnormal NMDAR expression and function have been observed in the brains of ASD patients [135,136] and several distinct mouse models of ASD [137,138,139,140,141,142]. In addition, NMDAR antagonists cause cognitive and behavioral changes that mimic those observed in ASD [131,143,144,145]. Additionally, lead is thought to dramatically increase the risk for ID in infants and children through antagonism of NMDARs [146,147,148,149,150]. Together, these data suggest that disturbances in NMDAR signaling have profound effects on neuronal development in humans, but the mechanisms that link NMDARs to developmental disorders are still being elucidated. Therefore, it is essential that we understand the links between glutamate signaling and synapse development.

Here, we have focused on the role of glutamate signaling via NMDARs in the control of presynaptic development. We have proposed that preNMDARs may contribute to glutamate-dependent presynaptic development. In the future, it will be interesting to know the extent to which glutamate and preNMDAR-dependent presynaptic development is disrupted in neurodevelopmental disorders.

## 8. Conclusions

It is now clear that NMDARs are found within presynaptic terminals of a variety of neurons, While these receptors have been demonstrated to regulate neurotransmitter release, their developmentally-regulated expression raises the question of what role these receptors play in both normal and abnormal neuronal development. Because molecular genetic techniques are not yet available to knockout only presynaptic receptors while leaving postsynaptic receptors intact, new methods for selectively controlling the expression or activity of presynaptic receptors on timescales of days to weeks will be needed to understand the developmental role of preNMDARs. This will likely require a better understanding of which proteins selectively interact with preNMDARs and/or control their expression and localization. In addition, it will be important to further delineate the downstream signaling pathways that mediate the effects of preNMDAR activation. While there is much to be done, this represents an exciting new direction for investigation into both NMDARs and presynaptic development.

## References

[1] Saito Y., Murakami F., Song W.J., Okawa K., Shimono K., Katsumaru H. (1992). Developing corticorubral axons of the cat form synapses on filopodial dendritic protrusions. Neurosci. Lett..

[2] Saito Y., Song W.J., Murakami F. (1997). Preferential termination of corticorubral axons on spine-like dendritic protrusions in developing cat. J. Neurosci..

[3] Ziv N.E., Smith S.J. (1996). Evidence for a role of dendritic filopodia in synaptogenesis and spine formation. Neuron.

[4] Dailey M.E., Smith S.J. (1996). The dynamics of dendritic structure in developing hippocampal slices. J. Neurosci..

[5] Fiala J.C., Feinberg M., Popov V., Harris K.M. (1998). Synaptogenesis via dendritic filopodia in developing hippocampal area CA1. J. Neurosci..

[6] Jontes J.D., Buchanan J., Smith S.J. (2000). Growth cone and dendrite dynamics in zebrafish embryos: Early events in synaptogenesis imaged *in vivo*. Nat. Neurosci..

[7] Ahmari S.E., Buchanan J., Smith S.J. (2000). Assembly of presynaptic active zones from cytoplasmic transport packets. Nat. Neurosci..

[8] Washbourne P., Bennett J.E., McAllister A.K. (2002). Rapid recruitment of NMDA receptor transport packets to nascent synapses. Nat. Neurosci..

[9] Shapira M., Zhai R.G., Dresbach T., Bresler T., Torres V.I., Gundelfinger E.D., Ziv N.E., Garner C.C. (2003). Unitary assembly of presynaptic active zones from Piccolo-Bassoon transport vesicles. Neuron.

[10] Kraszewski K., Mundigl O., Daniell L., Verderio C., Matteoli M., de Camilli P. (1995). Synaptic vesicle dynamics in living cultured hippocampal neurons visualized with CY3-conjugated antibodies directed against the lumenal domain of synaptotagmin. J. Neurosci..

[11] Sabo S.L., Gomes R.A., McAllister A.K. (2006). Formation of presynaptic terminals at predefined sites along axons. J. Neurosci..

[12] Cline H., Haas K. (2008). The regulation of dendritic arbor development and plasticity by glutamatergic synaptic input: A review of the synaptotrophic hypothesis. J. Physiol..

[13] Mozhayeva M.G., Sara Y., Liu X., Kavalali E.T. (2002). Development of vesicle pools during maturation of hippocampal synapses. J. Neurosci..

[14] Sceniak M.P., Berry C.T., Sabo S.L. (2012). Facilitation of neocortical presynaptic terminal development by NMDA receptor activation. Neural Dev..

[15] Berry C.T., Sceniak M.P., Zhou L., Sabo S.L. (2012). Developmental up-regulation of vesicular glutamate transporter-1 promotes neocortical presynaptic terminal development. PLoS ONE.

[16] Cull-Candy S.G., Leszkiewicz D.N. (2004). Role of distinct NMDA receptor subtypes at central synapses. Sci. STKE.

[17] Pachernegg S., Strutz-Seebohm N., Hollmann M. (2012). GluN3 subunit-containing NMDA receptors: Not just one-trick ponies. Trends Neurosci..

[18] Wang C.C., Held R.G., Chang S.C., Yang L., Delpire E., Ghosh A., Hall B.J. (2011). A critical role for GluN2B-containing NMDA receptors in cortical development and function. Neuron.

[19] Sanz-Clemente A., Nicoll R.A., Roche K.W. (2013). Diversity in NMDA receptor composition: Many regulators, many consequences. Neuroscientist.

[20] Monyer H., Sprengel R., Schoepfer R., Herb A., Higuchi M., Lomeli H., Burnashev N., Sakmann B., Seeburg P.H. (1992). Heteromeric NMDA receptors: Molecular and functional distinction of subtypes. Science.

[21] Larsen R.S., Corlew R.J., Henson M.A., Roberts A.C., Mishina M., Watanabe M., Lipton S.A., Nakanishi N., Perez-Otano I., Weinberg R.J. (2011). NR3A-containing NMDARs promote neurotransmitter release and spike timing-dependent plasticity. Nat. Neurosci..

[22] Mothet J.P., le Bail M., Billard J.M. (2015). Time and space profiling of NMDA receptor co-agonist functions. J. Neurochem..

[23] Van Horn M.R., Sild M., Ruthazer E.S. (2013). d-serine as a gliotransmitter and its roles in brain development and disease. Front. Cell. Neurosci..

[24] Le Bail M., Martineau M., Sacchi S., Yatsenko N., Radzishevsky I., Conrod S., Ait Ouares K., Wolosker H., Pollegioni L., Billard J.M. (2015). Identity of the NMDA receptor coagonist is synapse specific and developmentally regulated in the hippocampus. Proc. Natl. Acad. Sci. USA.

[25] Papouin T., Ladepeche L., Ruel J., Sacchi S., Labasque M., Hanini M., Groc L., Pollegioni L., Mothet J.P., Oliet S.H. (2012). Synaptic and extrasynaptic NMDA receptors are gated by different endogenous coagonists. Cell.

[26] Hashimoto A., Kumashiro S., Nishikawa T., Oka T., Takahashi K., Mito T., Takashima S., Doi N., Mizutani Y., Yamazaki T. (1993). Embryonic development and postnatal changes in free D-aspartate and D-serine in the human prefrontal cortex. J. Neurochem..

[27] Hashimoto A., Nishikawa T., Oka T., Takahashi K. (1993). Endogenous D-serine in rat brain: *N*-methyl-d-aspartate receptor-related distribution and aging. J. Neurochem..

[28] Hashimoto A., Oka T. (1997). Free D-aspartate and D-serine in the mammalian brain and periphery. Prog. Neurobiol..

[29] Puyal J., Martineau M., Mothet J.P., Nicolas M.T., Raymond J. (2006). Changes in D-serine levels and localization during postnatal development of the rat vestibular nuclei. J. Comp. Neurol..

[30] Verhage M., Maia A.S., Plomp J.J., Brussaard A.B., Heeroma J.H., Vermeer H., Toonen R.F., Hammer R.E., van den Berg T.K., Missler M. (2000). Synaptic assembly of the brain in the absence of neurotransmitter secretion. Science.

[31] Varoqueaux F., Sigler A., Rhee J.S., Brose N., Enk C., Reim K., Rosenmund C. (2002). Total arrest of spontaneous and evoked synaptic transmission but normal synaptogenesis in the absence of Munc13-mediated vesicle priming. Proc. Natl. Acad. Sci. USA.

[32] Han E.B., Stevens C.F. (2009). Development regulates a switch between post- and presynaptic strengthening in response to activity deprivation. Proc. Natl. Acad. Sci. USA.

[33] Wierenga C.J., Ibata K., Turrigiano G.G. (2005). Postsynaptic expression of homeostatic plasticity at neocortical synapses. J. Neurosci..

[34] Goold C.P., Nicoll R.A. (2010). Single-cell optogenetic excitation drives homeostatic synaptic depression. Neuron.

[35] Murthy V.N., Schikorski T., Stevens C.F., Zhu Y. (2001). Inactivity produces increases in neurotransmitter release and synapse size. Neuron.

[36] Ripley B., Otto S., Tiglio K., Williams M.E., Ghosh A. (2011). Regulation of synaptic stability by AMPA receptor reverse signaling. Proc. Natl. Acad. Sci. USA.

[37] Lee M.C., Yasuda R., Ehlers M.D. (2010). Metaplasticity at single glutamatergic synapses. Neuron.

[38] Ko J., Soler-Llavina G.J., Fuccillo M.V., Malenka R.C., Sudhof T.C. (2011). Neuroligins/LRRTMs prevent activity- and Ca^2+^/calmodulin-dependent synapse elimination in cultured neurons. J. Cell Biol..

[39] Ultanir S.K., Kim J.E., Hall B.J., Deerinck T., Ellisman M., Ghosh A. (2007). Regulation of spine morphology and spine density by NMDA receptor signaling *in vivo*. Proc. Natl. Acad. Sci. USA.

[40] Fremeau R.T., Kam K., Qureshi T., Johnson J., Copenhagen D.R., Storm-Mathisen J., Chaudhry F.A., Nicoll R.A., Edwards R.H. (2004). Vesicular glutamate transporters 1 and 2 target to functionally distinct synaptic release sites. Science.

[41] Bouwman J., Maia A.S., Camoletto P.G., Posthuma G., Roubos E.W., Oorschot V.M., Klumperman J., Verhage M. (2004). Quantification of synapse formation and maintenance *in vivo* in the absence of synaptic release. Neuroscience.

[42] Rao A., Craig A.M. (1997). Activity regulates the synaptic localization of the NMDA receptor in hippocampal neurons. Neuron.

[43] McKinney R.A., Luthi A., Bandtlow C.E., Gahwiler B.H., Thompson S.M. (1999). Selective glutamate receptor antagonists can induce or prevent axonal sprouting in rat hippocampal slice cultures. Proc. Natl. Acad. Sci. USA.

[44] Harms K.J., Craig A.M. (2005). Synapse composition and organization following chronic activity blockade in cultured hippocampal neurons. J. Comp. Neurol..

[45] Takada N., Yanagawa Y., Komatsu Y. (2005). Activity-dependent maturation of excitatory synaptic connections in solitary neuron cultures of mouse neocortex. Eur. J. Neurosci..

[46] Lazarevic V., Schone C., Heine M., Gundelfinger E.D., Fejtova A. (2011). Extensive remodeling of the presynaptic cytomatrix upon homeostatic adaptation to network activity silencing. J. Neurosci..

[47] Burrone J., O’Byrne M., Murthy V.N. (2002). Multiple forms of synaptic plasticity triggered by selective suppression of activity in individual neurons. Nature.

[48] Sutton M.A., Ito H.T., Cressy P., Kempf C., Woo J.C., Schuman E.M. (2006). Miniature neurotransmission stabilizes synaptic function via tonic suppression of local dendritic protein synthesis. Cell.

[49] Frank C.A., Kennedy M.J., Goold C.P., Marek K.W., Davis G.W. (2006). Mechanisms underlying the rapid induction and sustained expression of synaptic homeostasis. Neuron.

[50] Branco T., Staras K., Darcy K.J., Goda Y. (2008). Local dendritic activity sets release probability at hippocampal synapses. Neuron.

[51] Hou Q., Zhang D., Jarzylo L., Huganir R.L., Man H.Y. (2008). Homeostatic regulation of AMPA receptor expression at single hippocampal synapses. Proc. Natl. Acad. Sci. USA.

[52] Thiagarajan T.C., Lindskog M., Tsien R.W. (2005). Adaptation to synaptic inactivity in hippocampal neurons. Neuron.

[53] Aoto J., Nam C.I., Poon M.M., Ting P., Chen L. (2008). Synaptic signaling by all-trans retinoic acid in homeostatic synaptic plasticity. Neuron.

[54] Chubykin A.A., Atasoy D., Etherton M.R., Brose N., Kavalali E.T., Gibson J.R., Sudhof T.C. (2007). Activity-dependent validation of excitatory versus inhibitory synapses by neuroligin-1 versus neuroligin-2. Neuron.

[55] Ko H., Hofer S.B., Pichler B., Buchanan K.A., Sjostrom P.J., Mrsic-Flogel T.D. (2011). Functional specificity of local synaptic connections in neocortical networks. Nature.

[56] Kerschensteiner D., Morgan J.L., Parker E.D., Lewis R.M., Wong R.O. (2009). Neurotransmission selectively regulates synapse formation in parallel circuits *in vivo*. Nature.

[57] Atasoy D., Ertunc M., Moulder K.L., Blackwell J., Chung C., Su J., Kavalali E.T. (2008). Spontaneous and evoked glutamate release activates two populations of NMDA receptors with limited overlap. J. Neurosci..

[58] Sara Y., Bal M., Adachi M., Monteggia L.M., Kavalali E.T. (2011). Use-dependent AMPA receptor block reveals segregation of spontaneous and evoked glutamatergic neurotransmission. J. Neurosci..

[59] Autry A.E., Adachi M., Nosyreva E., Na E.S., Los M.F., Cheng P.F., Kavalali E.T., Monteggia L.M. (2011). NMDA receptor blockade at rest triggers rapid behavioural antidepressant responses. Nature.

[60] Alvarez V.A., Ridenour D.A., Sabatini B.L. (2007). Distinct structural and ionotropic roles of NMDA receptors in controlling spine and synapse stability. J. Neurosci..

[61] Colonnese M.T., Constantine-Paton M. (2006). Developmental period for *N-*methyl-d-aspartate (NMDA) receptor-dependent synapse elimination correlated with visuotopic map refinement. J. Comp. Neurol..

[62] Wang D.D., Kriegstein A.R. (2008). GABA regulates excitatory synapse formation in the neocortex via NMDA receptor activation. J. Neurosci..

[63] Ruthazer E.S., Li J., Cline H.T. (2006). Stabilization of axon branch dynamics by synaptic maturation. J. Neurosci..

[64] Luthi A., Schwyzer L., Mateos J.M., Gahwiler B.H., McKinney R.A. (2001). NMDA receptor activation limits the number of synaptic connections during hippocampal development. Nat. Neurosci..

[65] Ohno T., Maeda H., Murabe N., Kamiyama T., Yoshioka N., Mishina M., Sakurai M. (2010). Specific involvement of postsynaptic GluN2B-containing NMDA receptors in the developmental elimination of corticospinal synapses. Proc. Natl. Acad. Sci. USA.

[66] Liao D., Zhang X., O’Brien R., Ehlers M.D., Huganir R.L. (1999). Regulation of morphological postsynaptic silent synapses in developing hippocampal neurons. Nat. Neurosci..

[67] Li J., Erisir A., Cline H. (2011). *In vivo* time-lapse imaging and serial section electron microscopy reveal developmental synaptic rearrangements. Neuron.

[68] Nikonenko I., Jourdain P., Muller D. (2003). Presynaptic remodeling contributes to activity-dependent synaptogenesis. J. Neurosci..

[69] Bacci A., Coco S., Pravettoni E., Schenk U., Armano S., Frassoni C., Verderio C., de Camilli P., Matteoli M. (2001). Chronic blockade of glutamate receptors enhances presynaptic release and downregulates the interaction between synaptophysin-synaptobrevin-vesicle-associated membrane protein 2. J. Neurosci..

[70] Zhang W., Benson D.L. (2001). Stages of synapse development defined by dependence on F-actin. J. Neurosci..

[71] Takeichi M. (2007). The cadherin superfamily in neuronal connections and interactions. Nat. Rev. Neurosci..

[72] Sebeo J., Hsiao K., Bozdagi O., Dumitriu D., Ge Y., Zhou Q., Benson D.L. (2009). Requirement for protein synthesis at developing synapses. J. Neurosci..

[73] Aoki C., Venkatesan C., Go C.G., Mong J.A., Dawson T.M. (1994). Cellular and subcellular localization of NMDA-R1 subunit immunoreactivity in the visual cortex of adult and neonatal rats. J. Neurosci..

[74] Berretta N., Jones R.S. (1996). Tonic facilitation of glutamate release by presynaptic *N-*methyl-d-aspartate autoreceptors in the entorhinal cortex. Neuroscience.

[75] Fujisawa S., Aoki C. (2003). *In vivo* blockade of *N*-methyl-d-aspartate receptors induces rapid trafficking of NR2B subunits away from synapses and out of spines and terminals in adult cortex. Neuroscience.

[76] Corlew R., Wang Y., Ghermazien H., Erisir A., Philpot B.D. (2007). Developmental switch in the contribution of presynaptic and postsynaptic NMDA receptors to long-term depression. J. Neurosci..

[77] DeBiasi S., Minelli A., Melone M., Conti F. (1996). Presynaptic NMDA receptors in the neocortex are both auto- and heteroreceptors. Neuroreport.

[78] Charton J.P., Herkert M., Becker C.M., Schroder H. (1999). Cellular and subcellular localization of the 2B-subunit of the NMDA receptor in the adult rat telencephalon. Brain Res..

[79] McGuinness L., Taylor C., Taylor R.D., Yau C., Langenhan T., Hart M.L., Christian H., Tynan P.W., Donnelly P., Emptage N.J. (2010). Presynaptic NMDARs in the hippocampus facilitate transmitter release at theta frequency. Neuron.

[80] Siegel S.J., Brose N., Janssen W.G., Gasic G.P., Jahn R., Heinemann S.F., Morrison J.H. (1994). Regional, cellular, and ultrastructural distribution of *N*-methyl-d-aspartate receptor subunit 1 in monkey hippocampus. Proc. Natl. Acad. Sci. USA.

[81] Jourdain P., Bergersen L.H., Bhaukaurally K., Bezzi P., Santello M., Domercq M., Matute C., Tonello F., Gundersen V., Volterra A. (2007). Glutamate exocytosis from astrocytes controls synaptic strength. Nat. Neurosci..

[82] Aoki C., Fujisawa S., Mahadomrongkul V., Shah P.J., Nader K., Erisir A. (2003). NMDA receptor blockade in intact adult cortex increases trafficking of NR2A subunits into spines, postsynaptic densities, and axon terminals. Brain Res..

[83] Gill I., Droubi S., Giovedi S., Fedder K.N., Bury L.A., Bosco F., Sceniak M.P., Benfenati F., Sabo S.L. (2015). Presynaptic NMDA receptors—Dynamics and distribution in developing axons *in vitro* and *in vivo*. J. Cell Sci..

[84] Herkert M., Rottger S., Becker C.M. (1998). The NMDA receptor subunit NR2B of neonatal rat brain: Complex formation and enrichment in axonal growth cones. Eur. J. Neurosci..

[85] Ehlers M.D., Fung E.T., O’Brien R.J., Huganir R.L. (1998). Splice variant-specific interaction of the NMDA receptor subunit NR1 with neuronal intermediate filaments. J. Neurosci..

[86] Song A.H., Wang D., Chen G., Li Y., Luo J., Duan S., Poo M.M. (2009). A selective filter for cytoplasmic transport at the axon initial segment. Cell.

[87] Wang P.Y., Petralia R.S., Wang Y.X., Wenthold R.J., Brenowitz S.D. (2011). Functional NMDA receptors at axonal growth cones of young hippocampal neurons. J. Neurosci..

[88] Park H., Popescu A., Poo M.M. (2014). Essential role of presynaptic NMDA receptors in activity-dependent BDNF secretion and corticostriatal LTP. Neuron.

[89] Sjostrom P.J., Turrigiano G.G., Nelson S.B. (2003). Neocortical LTD via coincident activation of presynaptic NMDA and cannabinoid receptors. Neuron.

[90] Brasier D.J., Feldman D.E. (2008). Synapse-specific expression of functional presynaptic NMDA receptors in rat somatosensory cortex. J. Neurosci..

[91] Buchanan K.A., Blackman A.V., Moreau A.W., Elgar D., Costa R.P., Lalanne T., Tudor Jones A.A., Oyrer J., Sjostrom P.J. (2012). Target-specific expression of presynaptic NMDA receptors in neocortical microcircuits. Neuron.

[92] Rodriguez-Moreno A., Paulsen O. (2008). Spike timing-dependent long-term depression requires presynaptic NMDA receptors. Nat. Neurosci..

[93] Rodriguez-Moreno A., Kohl M.M., Reeve J.E., Eaton T.R., Collins H.A., Anderson H.L., Paulsen O. (2011). Presynaptic induction and expression of timing-dependent long-term depression demonstrated by compartment-specific photorelease of a use-dependent NMDA receptor antagonist. J. Neurosci..

[94] Banerjee A., Gonzalez-Rueda A., Sampaio-Baptista C., Paulsen O., Rodriguez-Moreno A. (2014). Distinct mechanisms of spike timing-dependent LTD at vertical and horizontal inputs onto L2/3 pyramidal neurons in mouse barrel cortex. Physiol. Rep..

[95] Cochilla A.J., Alford S. (1999). NMDA receptor-mediated control of presynaptic calcium and neurotransmitter release. J. Neurosci..

[96] Woodhall G., Evans D.I., Cunningham M.O., Jones R.S. (2001). NR2B-containing NMDA autoreceptors at synapses on entorhinal cortical neurons. J. Neurophysiol..

[97] Mameli M., Carta M., Partridge L.D., Valenzuela C.F. (2005). Neurosteroid-induced plasticity of immature synapses via retrograde modulation of presynaptic NMDA receptors. J. Neurosci..

[98] Lin H., Vicini S., Hsu F.C., Doshi S., Takano H., Coulter D.A., Lynch D.R. (2010). Axonal alpha7 nicotinic ACh receptors modulate presynaptic NMDA receptor expression and structural plasticity of glutamatergic presynaptic boutons. Proc. Natl. Acad. Sci. USA.

[99] Kunz P.A., Roberts A.C., Philpot B.D. (2013). Presynaptic NMDA receptor mechanisms for enhancing spontaneous neurotransmitter release. J. Neurosci..

[100] Bender V.A., Bender K.J., Brasier D.J., Feldman D.E. (2006). Two coincidence detectors for spike timing-dependent plasticity in somatosensory cortex. J. Neurosci..

[101] Nevian T., Sakmann B. (2006). Spine Ca^2+^ signaling in spike-timing-dependent plasticity. J. Neurosci..

[102] Min R., Nevian T. (2012). Astrocyte signaling controls spike timing-dependent depression at neocortical synapses. Nat. Neurosci..

[103] Rodriguez-Moreno A., Gonzalez-Rueda A., Banerjee A., Upton A.L., Craig M.T., Paulsen O. (2013). Presynaptic self-depression at developing neocortical synapses. Neuron.

[104] Corlew R., Brasier D.J., Feldman D.E., Philpot B.D. (2008). Presynaptic NMDA receptors: Newly appreciated roles in cortical synaptic function and plasticity. Neuroscientist.

[105] Isaac J.T., Nicoll R.A., Malenka R.C. (1995). Evidence for silent synapses: Implications for the expression of LTP. Neuron.

[106] Isaac J.T., Nicoll R.A., Malenka R.C. (1999). Silent glutamatergic synapses in the mammalian brain. Can. J. Physiol. Pharmacol..

[107] Pinheiro P.S., Mulle C. (2008). Presynaptic glutamate receptors: Physiological functions and mechanisms of action. Nat. Rev. Neurosci..

[108] Urban-Ciecko J., Wen J.A., Parekh P.K., Barth A.L. (2015). Experience-dependent regulation of presynaptic NMDARs enhances neurotransmitter release at neocortical synapses. Learn. Mem..

[109] Larsen R.S., Smith I.T., Miriyala J., Han J.E., Corlew R.J., Smith S.L., Philpot B.D. (2014). Synapse-specific control of experience-dependent plasticity by presynaptic NMDA receptors. Neuron.

[110] Huang L., Pallas S.L. (2001). NMDA antagonists in the superior colliculus prevent developmental plasticity but not visual transmission or map compression. J. Neurophysiol..

[111] Simon D.K., Prusky G.T., O’Leary D.D., Constantine-Paton M. (1992). *N*-methyl-d-aspartate receptor antagonists disrupt the formation of a mammalian neural map. Proc. Natl. Acad. Sci. USA.

[112] Ramoa A.S., Mower A.F., Liao D., Jafri S.I. (2001). Suppression of cortical NMDA receptor function prevents development of orientation selectivity in the primary visual cortex. J. Neurosci..

[113] Bury L.A., Sabo S.L. (2010). How it’s made: The synapse. Mol. Interv..

[114] Bourgeron T. (2009). A synaptic trek to autism. Curr. Opin. Neurobiol..

[115] Huttenlocher P.R., Dabholkar A.S. (1997). Regional differences in synaptogenesis in human cerebral cortex. J. Comp. Neurol..

[116] O’Roak B.J., Vives L., Fu W., Egertson J.D., Stanaway I.B., Phelps I.G., Carvill G., Kumar A., Lee C., Ankenman K. (2012). Multiplex Targeted Sequencing Identifies Recurrently Mutated Genes in Autism Spectrum Disorders. Science.

[117] O’Roak B.J., Vives L., Girirajan S., Karakoc E., Krumm N., Coe B.P., Levy R., Ko A., Lee C., Smith J.D. (2012). Sporadic autism exomes reveal a highly interconnected protein network of de novo mutations. Nature.

[118] Belmonte M.K., Allen G., Beckel-Mitchener A., Boulanger L.M., Carper R.A., Webb S.J. (2004). Autism and abnormal development of brain connectivity. J. Neurosci..

[119] Qiu S., Anderson C.T., Levitt P., Shepherd G.M. (2011). Circuit-specific intracortical hyperconnectivity in mice with deletion of the autism-associated Met receptor tyrosine kinase. J. Neurosci..

[120] Jamain S., Quach H., Betancur C., Rastam M., Colineaux C., Gillberg I.C., Soderstrom H., Giros B., Leboyer M., Gillberg C. (2003). Mutations of the X-linked genes encoding neuroligins NLGN3 and NLGN4 are associated with autism. Nat. Genet..

[121] Yan J., Oliveira G., Coutinho A., Yang C., Feng J., Katz C., Sram J., Bockholt A., Jones I.R., Craddock N. (2005). Analysis of the neuroligin 3 and 4 genes in autism and other neuropsychiatric patients. Mol. Psychiatry.

[122] Talebizadeh Z., Lam D.Y., Theodoro M.F., Bittel D.C., Lushington G.H., Butler M.G. (2006). Novel splice isoforms for NLGN3 and NLGN4 with possible implications in autism. J. Med. Genet..

[123] Geschwind D.H., Levitt P. (2007). Autism spectrum disorders: Developmental disconnection syndromes. Curr. Opin. Neurobiol..

[124] Helmeke C., Ovtscharoff W., Poeggel G., Braun K. (2001). Juvenile emotional experience alters synaptic inputs on pyramidal neurons in the anterior cingulate cortex. Cereb. Cortex.

[125] Poeggel G., Helmeke C., Abraham A., Schwabe T., Friedrich P., Braun K. (2003). Juvenile emotional experience alters synaptic composition in the rodent cortex, hippocampus, and lateral amygdala. Proc. Natl. Acad. Sci. USA.

[126] Breslau N., Schultz L., Peterson E. (1995). Sex differences in depression: A role for preexisting anxiety. Psychiatry Res..

[127] Parker G., Wilhelm K., Mitchell P., Austin M.P., Roussos J., Gladstone G. (1999). The influence of anxiety as a risk to early onset major depression. J. Affect. Disord..

[128] Weissman M.M., Wickramaratne P., Nomura Y., Warner V., Verdeli H., Pilowsky D.J., Grillon C., Bruder G. (2005). Families at high and low risk for depression: A 3-generation study. Arch. Gen. Psychiatry.

[129] Leonardo E.D., Hen R. (2008). Anxiety as a developmental disorder. Neuropsychopharmacology.

[130] Endele S., Rosenberger G., Geider K., Popp B., Tamer C., Stefanova I., Milh M., Kortum F., Fritsch A., Pientka F.K. (2010). Mutations in GRIN2A and GRIN2B encoding regulatory subunits of NMDA receptors cause variable neurodevelopmental phenotypes. Nat. Genet..

[131] Tarabeux J., Kebir O., Gauthier J., Hamdan F.F., Xiong L., Piton A., Spiegelman D., Henrion E., Millet B., Fathalli F. (2011). Rare mutations in *N*-methyl-d-aspartate glutamate receptors in autism spectrum disorders and schizophrenia. Transl. Psychiatry.

[132] Autism Genome Project C., Szatmari P., Paterson A.D., Zwaigenbaum L., Roberts W., Brian J., Liu X.Q., Vincent J.B., Skaug J.L., Thompson A.P. (2007). Mapping autism risk loci using genetic linkage and chromosomal rearrangements. Nat. Genet..

[133] Gai X., Xie H.M., Perin J.C., Takahashi N., Murphy K., Wenocur A.S., D’Arcy M., O’Hara R.J., Goldmuntz E., Grice D.E. (2012). Rare structural variation of synapse and neurotransmission genes in autism. Mol. Psychiatry.

[134] Hamdan F.F., Gauthier J., Araki Y., Lin D.T., Yoshizawa Y., Higashi K., Park A.R., Spiegelman D., Dobrzeniecka S., Piton A. (2011). Excess of de novo deleterious mutations in genes associated with glutamatergic systems in nonsyndromic intellectual disability. Am. J. Hum. Genet..

[135] Purcell A.E., Jeon O.H., Zimmerman A.W., Blue M.E., Pevsner J. (2001). Postmortem brain abnormalities of the glutamate neurotransmitter system in autism. Neurology.

[136] Blatt G.J., Fitzgerald C.M., Guptill J.T., Booker A.B., Kemper T.L., Bauman M.L. (2001). Density and distribution of hippocampal neurotransmitter receptors in autism: An autoradiographic study. J. Autism Dev. Disord..

[137] Eadie B.D., Cushman J., Kannangara T.S., Fanselow M.S., Christie B.R. (2012). NMDA receptor hypofunction in the dentate gyrus and impaired context discrimination in adult Fmr1 knockout mice. Hippocampus.

[138] Maliszewska-Cyna E., Bawa D., Eubanks J.H. (2010). Diminished prevalence but preserved synaptic distribution of *N*-methyl-d-aspartate receptor subunits in the methyl CpG binding protein 2(MeCP2)-null mouse brain. Neuroscience.

[139] Bangash M.A., Park J.M., Melnikova T., Wang D., Jeon S.K., Lee D., Syeda S., Kim J., Kouser M., Schwartz J. (2011). Enhanced polyubiquitination of Shank3 and NMDA receptor in a mouse model of autism. Cell.

[140] Rinaldi T., Kulangara K., Antoniello K., Markram H. (2007). Elevated NMDA receptor levels and enhanced postsynaptic long-term potentiation induced by prenatal exposure to valproic acid. Proc. Natl. Acad. Sci. USA.

[141] Etherton M., Foldy C., Sharma M., Tabuchi K., Liu X., Shamloo M., Malenka R.C., Sudhof T.C. (2011). Autism-linked neuroligin-3 R451C mutation differentially alters hippocampal and cortical synaptic function. Proc. Natl. Acad. Sci. USA.

[142] Sceniak M.P., Lang M., Enomoto A.C., James Howell C., Hermes D.J., Katz D.M. (2015). Mechanisms of functional hypoconnectivity in the medial prefrontal cortex of Mecp2 null mice. Cereb. Cortex.

[143] Choudhury P.R., Lahiri S., Rajamma U. (2012). Glutamate mediated signaling in the pathophysiology of autism spectrum disorders. Pharmacol. Biochem. Behav..

[144] Chez M.G., Burton Q., Dowling T., Chang M., Khanna P., Kramer C. (2007). Memantine as adjunctive therapy in children diagnosed with autistic spectrum disorders: An observation of initial clinical response and maintenance tolerability. J. Child Neurol..

[145] Kron M., Howell C.J., Adams I.T., Ransbottom M., Christian D., Ogier M., Katz D.M. Brain activity mapping in Mecp2 mutant mice reveals functional deficits in forebrain circuits, including key nodes in the default mode network, that are reversed with ketamine treatment. J. Neurosci..

[146] Alkondon M., Costa A.C., Radhakrishnan V., Aronstam R.S., Albuquerque E.X. (1990). Selective blockade of NMDA-activated channel currents may be implicated in learning deficits caused by lead. FEBS Lett..

[147] Gavazzo P., Zanardi I., Baranowska-Bosiacka I., Marchetti C. (2008). Molecular determinants of Pb^2+^ interaction with NMDA receptor channels. Neurochem. Int..

[148] Neal A.P., Worley P.F., Guilarte T.R. (2011). Lead exposure during synaptogenesis alters NMDA receptor targeting via NMDA receptor inhibition. Neurotoxicology.

[149] Omelchenko I.A., Nelson C.S., Allen C.N. (1997). Lead inhibition of *N-*methyl-d-aspartate receptors containing NR2A, NR2C and NR2D subunits. J. Pharmacol. Exp. Ther..

[150] Ujihara H., Albuquerque E.X. (1992). Developmental change of the inhibition by lead of NMDA-activated currents in cultured hippocampal neurons. J. Pharmacol. Exp. Ther..

